# Supplementary material for: Identification of Key Ferroptosis-Related Genes in the Peripheral Blood of Patients with Relapsing-Remitting Multiple Sclerosis and Its Diagnostic Value
Source: Int J Mol Sci. 2023 Mar 29;24(7):6399. doi: 10.3390/ijms24076399 (PMC10094542; doi:10.3390/ijms24076399)
Supplement: Supplementary file 1 [file ijms-24-06399-s001.zip › Supplementary File 4.pdf]

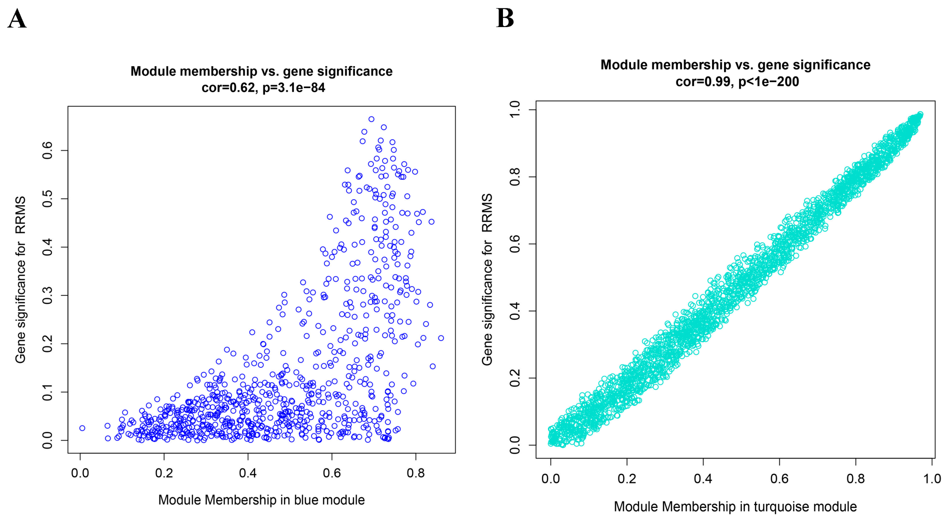

Supplementary File 4. Scatter plots were created to show the correlation between module membership and eigengenes in the blue and turquoise modules, and the gene significance for Relapsing-remitting multiple sclerosis.
